# Licochalcone a Induces ROS-Mediated Apoptosis through TrxR1 Inactivation in Colorectal Cancer Cells

**DOI:** 10.1155/2020/5875074

**Published:** 2020-05-27

**Authors:** Peng Wu, Ting Yu, Jun Wu, Junfeng Chen

**Affiliations:** ^1^Department of Clinical Laboratory, Renmin Hospital of Wuhan University, Wuhan 430060, China; ^2^Wuhan Mental Health Center, Wuhan 430012, China; ^3^Department of Pharmacy, Wuhan Jinyin Tan Hospital, Wuhan 430023, China; ^4^Department of Clinical Laboratory, HwaMei Hospital, University of Chinese Academy of Sciences, Ningbo 315010, China; ^5^Ningbo Institute of Life and Health Industry, University of Chinese Academy of Sciences, Ningbo 315010, China

## Abstract

Licochalcone A (LCA) exhibited anticancer activity through modulating reactive oxygen species (ROS) levels in some cancer cells and has been evidenced to suppress colorectal cancer (CRC) formation and progression. However, whether LCA mediates the progression of CRC by regulating ROS production remains unclear. To address this, HCT-116 cells were treated with LCA, resulting in G0/G1 phase arrest, apoptosis, and high ROS generation, which were attenuated by N-acetyl-L-cysteine, a ROS inhibitor. In addition, LCA suppressed the expression of thioredoxin reductase 1 (TrxR1) in HCT-116 cells, leading to high ROS levels and apoptosis. Moreover, LCA administration combined with TrxR1 inhibition further enhanced the production of ROS and apoptosis in HCT-116 cells compared to LCA administration or TrxR1 inhibition alone. These results demonstrated that LCA might enhance the production of ROS by targeting TrxR1, leading to apoptosis in HCT-116 cells, which provides potential insight for the interventional treatment of CRC.

## 1. Introduction

Colorectal cancer (CRC) is a major cause of tumor-related deaths because of its spreading capability and metastatic characteristic [1, 2]. Surgery is the main treatment for CRC, but its efficacy is limited by diagnosis in the middle and advanced stages and high recurrence rate after operation. Chemotherapy, an important part of comprehensive treatment, plays a critical role in relieving cancer symptoms and prolonging patient survival. However, traditional chemotherapeutic drugs such as 5-fluorouracil and cisplatin are often limited by drug resistance and side effects. Natural products and their analogs are promising anticancer drugs because of their remarkable effects, low toxicity, and reduced side effects.

*Glycyrrhiza uralensis* is widely used in traditional Chinese medicine. Licochalcone A (LCA) is a flavonoid extracted from glycyrrhiza glabra and glycyrrhiza inflate with pharmacological activities such as anticancer, anti-inflammatory, and antioxidation properties [3–5]. Accumulating evidence has demonstrated that LCA exhibited anticancer activity through modulating reactive oxygen species (ROS) levels in cancer cells. Hao et al. suggested that LCA enhanced BGC-823 human gastric cancer cell apoptosis by activating ROS-mediated mitogen-activated protein kinase and PI3K/AKT signaling pathways [6]. Choi et al. found that LCA induced apoptosis through endoplasmic reticulum stress of HepG2 human hepatocellular carcinoma cells by regulating ROS-dependent signaling pathways [7]. Previous research has demonstrated that LCA attenuated the expression of inflammatory mediators, in turn modifying the tumor microenvironment and therefore suppressing CRC formation and progression [8]. However, whether LCA mediates the progression of CRC by regulating ROS production and the underlying targets remains unclear.

In this work, the human CRC cell line HCT-116 was treated by LCA, resulting in apoptosis and high ROS production. Furthermore, the effect of ROS production induced by LCA in HCT-116 cells and the underlying target were investigated.

## 2. Materials and Methods

### 2.1. Cell Culture and Treatment

The human CRC cell line HCT-116 was supplied by the Shanghai Institutes for Biological Sciences, Chinese Academy of Science. HCT-116 cells were cultured in McCoy's 5A medium (Gibco BRL, Gaithersburg, MD, USA) supplemented with 10% fetal bovine serum (FBS, Gibco), and normal human colon epithelial cells Ncm460 were cultured in Dulbecco's modified Eagle medium (Hyclone, UT, USA) supplemented with 10% FBS. Both HCT-116 and Ncm460 cells were maintained in a humidified incubator at 37°C with 5% CO_2_. Cells at the logarithmic phase were harvested and treated with LCA (Aladdin, Shanghai, China) at different concentrations (2.5, 5, 10, 20, 40, 80, 120, and 160 *μ*M) for 24 h (a stock solution of LCA was prepared in dimethyl sulfoxide at 40 mM, followed by dilution into different concentration accordingly). The untreated cells served as the control. The cell viability was detected using the 3-(4,5-dimethylthiazol-2-yl)-2,5-diphenyltetrazolium bromide (MTT) assay.

Next, HCT-116 cells in the logarithmic growth phase were harvested and treated with different concentrations of LCA (10, 20, or 40 *μ*M) for 24 h, and untreated cells served as controls. Flow cytometry was performed to evaluate cell cycle and apoptosis. In addition, the expression of proteins related to cell cycle progression and apoptosis was detected using western blot.

Then, HCT-116 cells in the logarithmic growth phase were treated with 40 *μ*M LCA for 0.5, 1, 2, 4, 8, or 12 h, and ROS production was examined using flow cytometry. Cells were also treated with 5 mM N-acetyl-L-cysteine (Selleck Houston, Texas, USA), a ROS inhibitor, for 1 h and then treated with 40 *μ*M LCA for 12 h. Untreated cells served as a control. ROS production, cell cycle progression, and apoptosis were evaluated using flow cytometry. Western blot was carried out to analyze the expression of relevant proteins.

### 2.2. MTT Assay

Ncm460 and HCT-116 cells were seeded into a 96-well plate (5 × 103 cells/well) and maintained at 37°C with 5% CO_2_ until the cells adhered. After treatment, 20 *μ*l of MTT at a concentration of 5 mg/ml was added to each well for 4 h, and 150 *μ*l of dimethyl sulfoxide (Sigma, MO, USA) was then added to each well. The plate was shaken for 10 min, and the absorbance was evaluated using a Multiskan FC apparatus (Thermo, Waltham, MA, USA) at 490 nm.

### 2.3. Flow Cytometry

For cell cycle evaluation, 1 × 106 harvested HCT-116 cells were fixed in absolute ethyl alcohol at -20°C for 24 h, followed by centrifugation at 3,000 × g for 30 s. After two washes with precooled phosphate-buffered saline (Bioswamp, Myhalic Biotechnology Co., Ltd., Wuhan, China), the cells were resuspended in 100 *μ*l of 1 mg/ml RNase A, stained with 400 *μ*l of 50 *μ*l/ml propidium iodide (PI) for 10 min in the dark, and subjected to flow cytometry (Beckman Coulter, Brea, CA, USA). For apoptosis, the Annexin V-fluorescein (FITC)/PI apoptosis detection assay (BD, Shanghai, China) was performed according to the manufacturer's instruction. The collected cells (1 × 105) were resuspended in 200 *μ*l of binding buffer after centrifugation at 1,000 × g, 4°C for 5 min (three times) and stained with 10 *μ*l of Annexin V-FITC and 10 *μ*l of PI in the dark for 30 min at 4°C. Then, the apoptosis ratio was measured using an FC500 MCL apparatus (Beckman Coulter). For ROS detection, harvested cells (1 × 106 cells) were mixed with diluted 2′,7′-dichlorofluorescin diacetate (DCFH-DA) fluoroprobes (10 *μ*mol/l, Bioswamp) for 20 min at 37°C with gentle shaking every 4 min. Nonattached DCFH-DA was removed by washing with serum-free medium, and the cells were subjected to flow cytometry (ACEA Biosciences, San Diego, CA, USA).

### 2.4. Western Blot

Total protein content was extracted from 3 × 105 cells in each group using radioimmunoprecipitation assay lysis buffer (Bioswamp) and quantified using a bicinchoninic acid kit (Bioswamp) following the manufacturer's protocol. 20 *μ*g of proteins were separated by sodium dodecyl sulfate-polyacrylamide gel electrophoresis and transferred onto polyvinylidene fluoride membranes (Millipore, MA, USA). After blocking with 5% skim milk for 2 h at room temperature, the membranes were incubated with primary antibodies against CDC2 (Bioswamp, PAB30052, 1 : 1,000), B-cell lymphoma-2 (Bcl-2, Bioswamp, PAB30042, 1 : 1,000), Bcl-2-associated X (Bax, Bioswamp, PAB30040, 1 : 1,000), nuclear factor erythroid-2-related factor 2 (Nrf2, Bioswamp, PAB30175, 1 : 1,000), TrxR1 (abcam, ab124954, 1 : 5,000), apoptosis signal regulating kinase 1 (ASK1, Bioswamp, PAB36297-P, 1 : 1,000), phosphorylated (p)-ASK1 (abcam, ab47304, 1 : 1,000), and GAPDH (Bioswamp, PAB36269, 1 : 1,000) overnight at 4°C. After washing, the membranes were incubated with horseradish peroxidase-labeled goat antirabbit IgG secondary antibody (Bioswamp, PAB160011, 1 : 20,000) for 1 h at room temperature and visualized using a Tanon-5200 apparatus (Tanon, Shanghai, China). The band gray values were read using the TANON GIS software (Tanon). GAPDH acted as the internal reference.

### 2.5. Cell Transfection and Treatment

The TrxR1 short hairpin RNA (shRNA, sequence: 5′-GCTGCAGACGAAAGGCAAGAA-3′) and its corresponding negative control (sh-NC, sequence: 5′-AAGCTTCGATGCTTAGTAAGC-3′) were subcloned into the pSICOR vector (Addgene, Cambridge, MA, USA). The constructed plasmid vectors (sh-TrxR1 or sh-NC) were transfected into HCT-116 cells using Lipofectamine 2000 (Invitrogen, Carlsbad, CA, USA). Cells were collected 24 hours after transfection for quantitative reverse transcription polymerase chain reaction (qRT-PCR) and western blot. The transfected or untransfected cells were then administered with or without 40 *μ*l of LCA for 24 h and subjected to flow cytometry and western blot, as mentioned previously.

### 2.6. qRT-PCR

TRIzol (Ambion, Texas, USA) was used to extract total RNA from the transfected HCT-116 cells. The extracted RNA was then reversed-transcribed into cDNA, which was subjected to amplification using a CFX-CONNECT 96 apparatus (Bio-Rad, Hercules, CA, USA). The primers were: TrxR1 forward, 5′-AGGAGGGCAGACTTCAAAA-3′, reverse, 5′-CCATCTAGTTCCAAGAGGG-3′; GAPDH forward, 5′-CCACTCCTCCACCTTTG-3′, reverse, 5′-CACCACCCTGTTGCTGT-3′. GAPDH served as an endogenous control. The 2^-△△Ct^ method was utilized for the calculation of relative mRNA levels [9].

### 2.7. Statistical Analysis

Data are presented as the mean ± standard deviation (SD). Differences between more than two groups were analyzed using one-way analysis of variance followed by Tukey or least significant difference test. *P* < 0.05 was considered to be statistically significant.

## 3. Results

### 3.1. LCA Attenuated Cell Viability and Stimulated G0/G1 Phase Arrest and Apoptosis in a Dose-Dependent Manner in HCT-116 Cells

**Figure 1:**
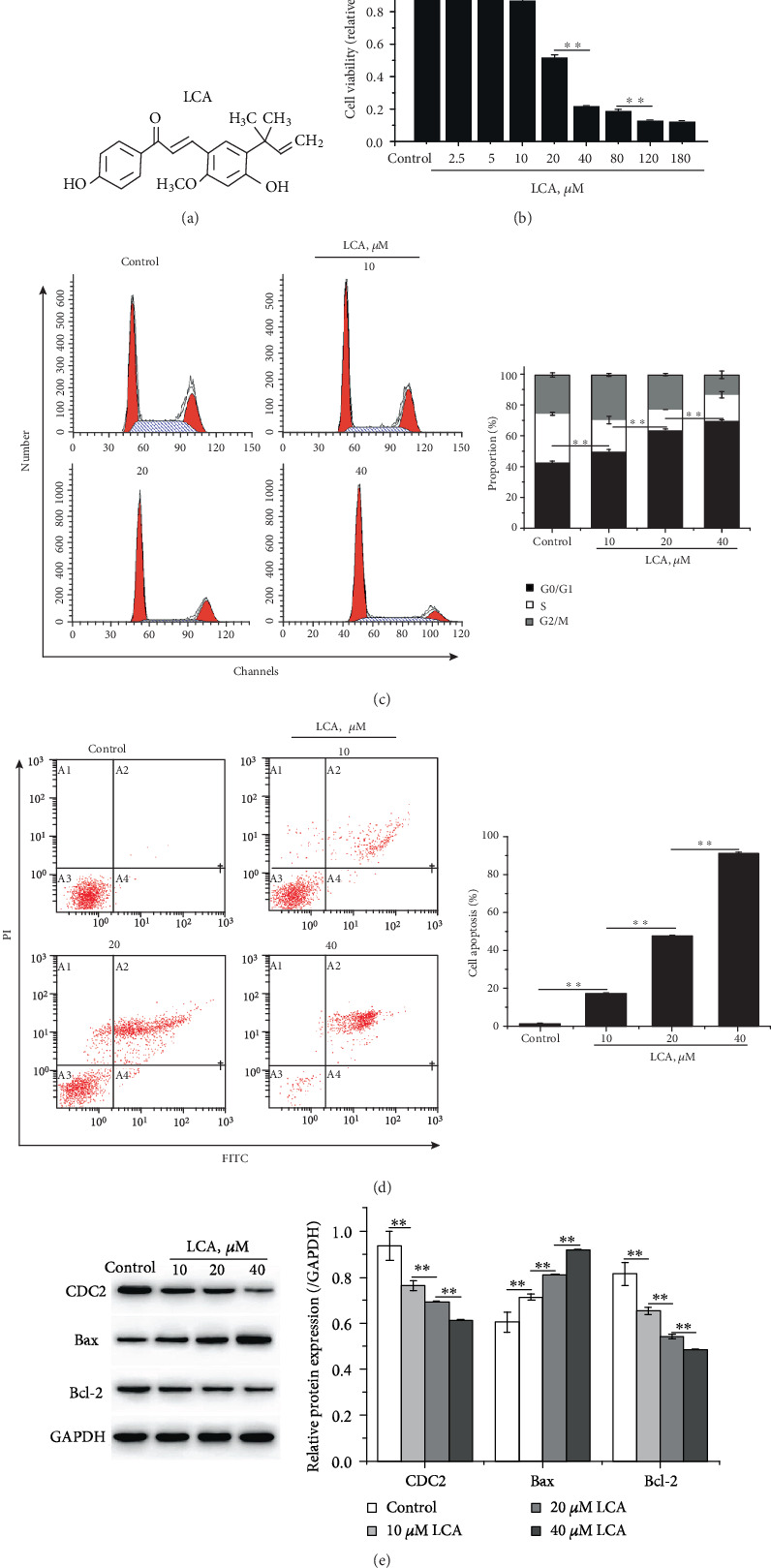
LCA attenuated the viability and promoted apoptosis of HCT-116 cells in a dose-dependent manner. (a) The chemical structure of LCA. (b) Cell viability was measured using MTT assay. (c) Cell cycle was detected by flow cytometry. (d) Apoptosis was evaluated using flow cytometry. (e) Apoptosis- and cell cycle-related proteins were detected by western blot. Data are expressed as the mean ± SD (*n* = 3). ∗∗*P* < 0.01.

Figure 1(a) illustrates the chemical structure of LCA. The effect of LCA on cell viability, cell cycle, and apoptosis was evaluated in HCT-116 cells using MTT assay and flow cytometry. MTT assay demonstrated that the survival of cells treated with LCA (40 *μ*M) for 24 h remained at over 80% of that of the control cells, indicating the little toxicity of LCA for Ncm460 cells (Figure S1), and that low concentrations of LCA increased HCT-116 cell viability, whereas high concentrations of LCA reduced cell viability in a dose-dependent manner (*P* < 0.01, Figure 1(b)). Comprehensively considering the cell viability at different concentrations of LCA and the purpose of this experiment, 10, 20, and 40 *μ*M were selected for subsequent experiments. Flow cytometry indicated that LCA contributed to G0/G1 phase arrest and apoptosis of HCT-116 cells in a dose-dependent manner (*P* < 0.01, Figures 1(c) and 1(d)). The results of western blot suggested that LCA suppressed the protein expression of CDC2 and Bcl-2 while promoting that of Bax compared to those in control cells (*P* < 0.01, Figure 1(e)).

### 3.2. LCA Stimulated G0/G1 Arrest and Apoptosis in HCT-116 Cells by Elevating ROS Level.

**Figure 2:**
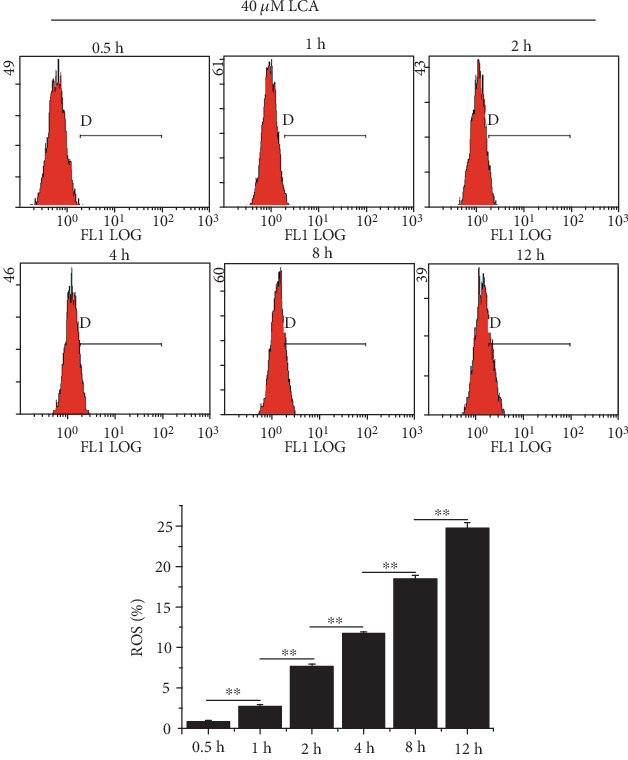
LCA increased ROS production in HCT-116 cells in a time-dependent manner. ROS level was measured using flow cytometry after treatment with 40 *μ*M LCA for different time periods.

**Figure 3:**
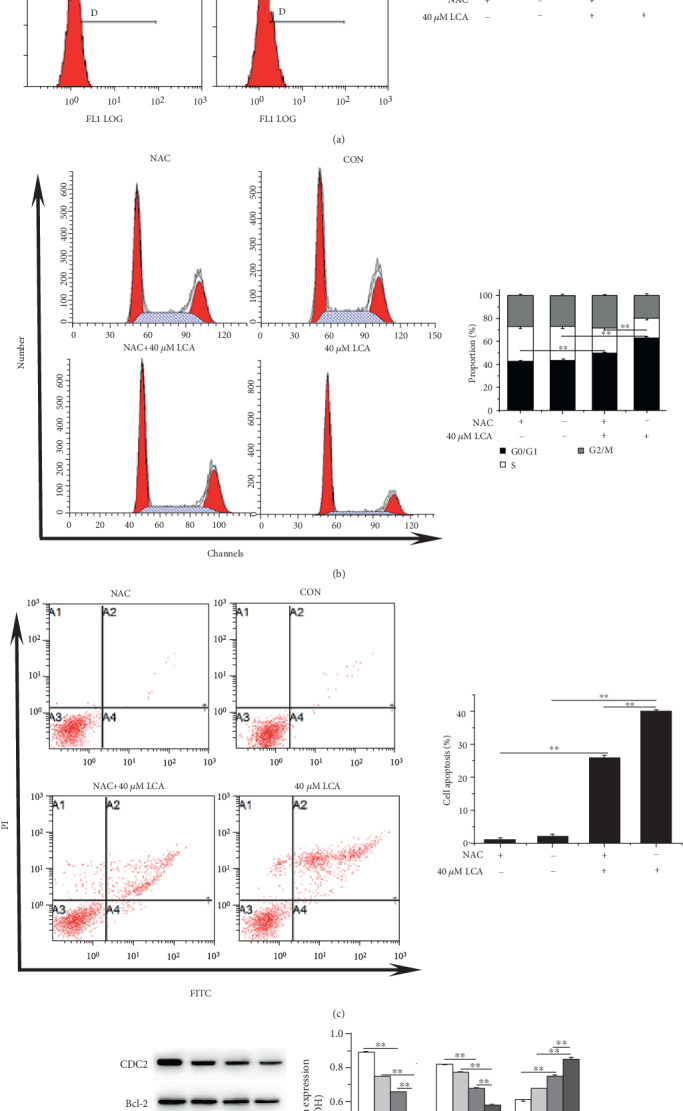
LCA induced HCT-116 cell apoptosis by increasing ROS production. (a) ROS level was measured using flow cytometry. (b) Cell cycle was detected using flow cytometry. (c) Apoptosis was evaluated using flow cytometry. (d) Apoptosis- and cell cycle-related proteins were detected by western blot. Data are expressed as the mean ± SD (*n* = 3). ∗∗*P* < 0.01.

**Figure 4:**
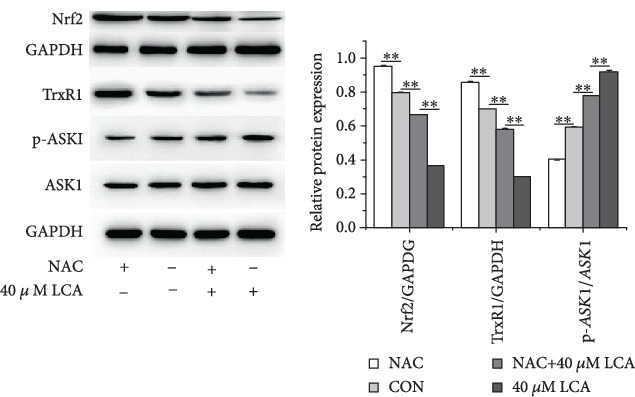
LCA attenuated the activation of TrxR1 signaling pathway in HCT-116 cells. The protein expression of Nrf2, TrxR1, p-ASK1, and ASK1 was measured using western blot. Data are expressed as the mean ± SD (*n* = 3). ∗∗*P* < 0.01.

As shown in Figure 2, LCA at 40 *μ*M enhanced ROS levels in HCT-116 cells in a time-dependent manner and the addition of NAC inhibited LCA-induced ROS production (*P* < 0.01, Figure 3(a)). Then cell cycle progression, apoptosis, and associated proteins were evaluated. The results indicated that NAC attenuated LCA-induced G0/G1 phase arrest and apoptosis in HCT-116 cells (*P* < 0.01, Figures 3(b) and 3(c)). In addition, NAC increased the protein expression of CDC2, and Bcl-2 was decreased by LCA, while Bax showed the opposite tendency (*P* < 0.01, Figure 3(d)). Furthermore, LCA decreased the protein expression of Nrf2, TrxR1, and increased the expression of p-ASK1 in HCT-116 cells compared to that in cells treated with or without NAC (*P* < 0.01, Figure 4). Collectively, these results indicate that LCA contributes to cell G0/G1 phase arrest and apoptosis in HCT-116 cells by upregulating ROS levels.

### 3.3. LCA Upregulated ROS Levels through Inactivation of TrxR1 Pathway in HCT-116 Cells

**Figure 5:**
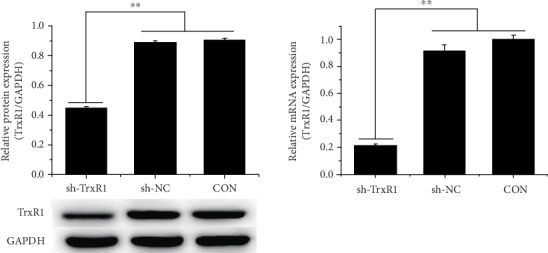
The expression of TrxR1 was inhibited by sh-TrxR1 transfection in HCT-116 cells. Protein and mRNA expression of TrxR1 was measured using western blot and qRT-PCR, respectively. Data are expressed as the mean ± SD (*n* = 3). ∗∗*P* < 0.01.

**Figure 6:**
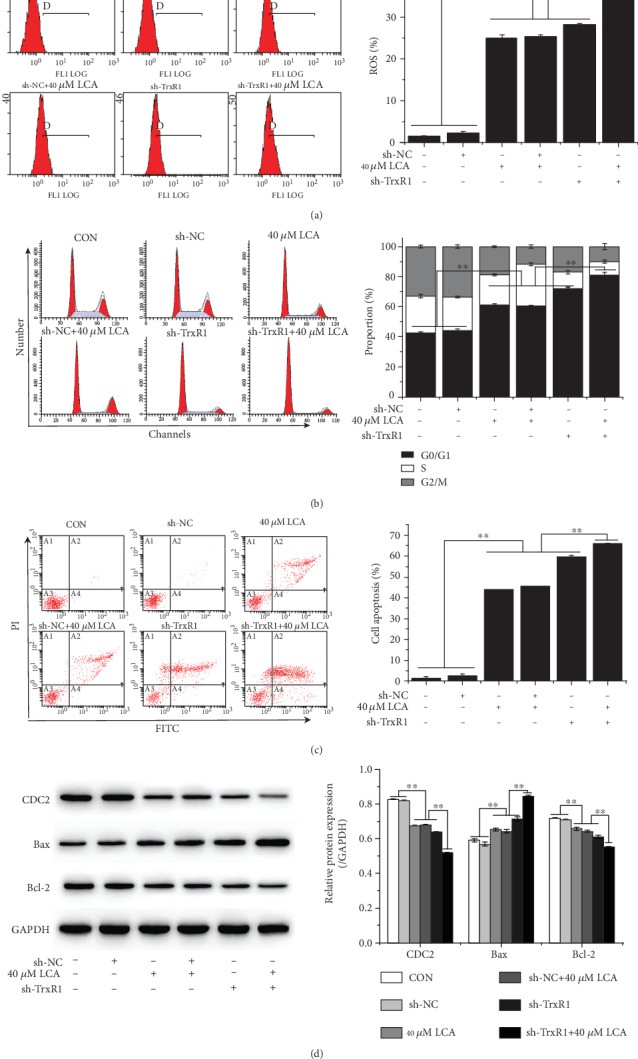
LCA upregulated ROS level and promoted apoptosis through inactivation of the TrxR1 pathway in HCT-116 cells. (a) ROS level was measured using flow cytometry. (b) Cell cycle was detected by flow cytometry. (c) Apoptosis was evaluated using flow cytometry. (d) Apoptosis- and cell cycle-related proteins were detected by western blot. Data are expressed as the mean ± SD (*n* = 3). ∗∗*P* < 0.01.

**Figure 7:**
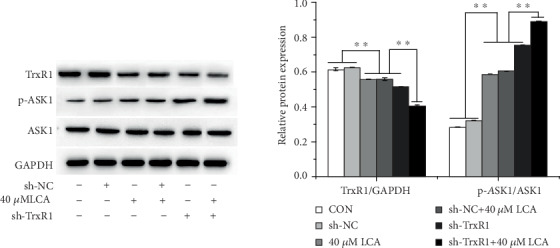
Combination of LCA and sh-TrxR1 attenuated the activation of TrxR1 signaling in HCT-116 cells. The protein expression of TrxR1, p-ASK1, and ASK1 was measured using western blot. Data are expressed as the mean ± SD (*n* = 3). ∗∗*P* < 0.01.

To investigate whether the LCA-induced increase in ROS level is associated with the TrxR1 pathway, the expression of TrxR1 in HCT-116 cells was inhibited by sh-TrxR1 transfection (Figure 5), and the transfected cells were treated with LCA for 24 h. Flow cytometry demonstrated that TrxR1 inhibition enhanced ROS production, promoted apoptosis, and resulted in G0/G1 phase arrest of HCT-116 cells compared to those of control and sh-NC-transfected cells (*P* < 0.01, Figures 6(a)–6(c)). In addition, TrxR1 inhibition decreased the protein expression of CDC2 and Bcl-2 and increased that of Bax (Figure 6(d)). Compared to LCA treatment or sh-TrxR1 transfection alone, the combination of LCA treatment with sh-TrxR1 transfection increased ROS level, apoptosis, and G0/G1 phase arrest of HCT-116 cells while downregulating CDC2 and Bcl-2 and upregulating Bax (*P* < 0.01, Figure 5). Additionally, combined LCA treatment with sh-TrxR1 transfection reduced the protein expression of TrxR1 and increased the protein expression of p-ASK1 to a greater extent than LCA treatment or sh-TrxR1 transfection alone in HCT-116 cells (*P* < 0.01, respectively, Figure 7). Taken together, LCA-induced ROS upregulation in HCT-116 cells might be associated with TrxR1 inactivation.

## 4. Discussion

Herein, we provided the first evidence supporting that LCA stimulated ROS production in HCT-116 cells, in turn promoting G0/G1 phase arrest. This was demonstrated by the low expression of CDC2, a cyclin-dependent kinase, whose inhibition leads to G0/G1 phase arrest [10]. Apoptosis was indicated by the low expression of Bcl-2, an antiapoptotic protein, and the high expression of Bax, a proapoptotic protein [11], which was attenuated after ROS inhibition by the antioxidant NAC. Previously, Niu et al. reported that LCA led to high production of ROS, thereby inducing autophagy in human hepatocellular carcinoma cells, and inhibition of LCA-induced ROS by NAC participated in LCA-mediated apoptosis [12]. Wang et al. found that LCA played an anticancer role in human hepatoma cells through enhancing G2/M phase arrest and apoptosis [13]. These findings suggested the different regulatory functions of LCA in different cancer cells.

Oxidative stress is involved in regulating the biological behavior of cancer cells. Although normal physiological ROS generation plays an important role in maintaining multiple cellular functions, abnormally high expression of ROS disrupts the balance between oxidation and antioxidation. This in turn results in oxidative stress, which can lead to cell death or damage [14, 15]. Furthermore, the underlying target of LCA-induced ROS production in HCT-116 cells was investigated. The thioredoxin (Trx) system has been verified to participate in the regulation of redox balance in cancer cells, which contains Trx, Trx reductase (TrxR), and Trx-interacting proteins [16, 17]. TrxR is the only known protein capable of reducing Trx, the inhibition of which results in redox dysfunction in some cells and leads to increased ROS production [18]. There are at least three TrxR isoforms, namely cytosolic TrxR1, mitochondrial TrxR2, and testis-specific TrxR3 [18]. TrxR1 is present in most tissues and is denoted as the predominant TrxR of the three [19]. Previous research found that the inhibition of TrxR1 via auranofin, an inhibitor of TrxR1, induced CRC cell mortality through increasing ROS production [20]. In this work, LCA administration decreased the expression of TrxR1 in HCT-116 cells and increased that of ASK1, which can be activated by ROS and in turn induces cell apoptosis through regulating downstream signaling pathways [20]. In addition, TrxR1 inhibition attenuated the reduction of Trx, whose reduction state inhibits the expression of ASK1, thereby suppressing cell apoptosis [21]. Therefore, we hypothesized that LCA may induce ROS production by targeting TrxR1 in HCT-116 cells. The results demonstrated that TrxR1 inhibition promoted ROS production, ASK1 expression, and apoptosis in HCT-116 cells. In addition, combined LCA treatment with TrxR1 inhibition further downregulated TrxR1 and promoted ROS production, ASK1 expression, and apoptosis in HCT-116 cells, confirming our hypothesis.

The present work also demonstrated that LCA decreased the expression of Nrf2, a redox-sensitive transcription factor that modulates various antioxidant genes via an antioxidant response element [22]. Evidences demonstrated that Nrf2 is a major regulator of TrxR1 [23]. Nrf2 activation promoted the expression of TrxR1, in turn protecting C6 glial cells from hydrogen peroxide-induced apoptosis and oxidative stress [24]. Thus, we speculate that the downregulation of TrxR1 induced by LCA might be associated with Nrf2, which need to be further verified.

Taken together, we present the first evidence revealing that LCA enhances the production of ROS by targeting TrxR1, leading to HCT-116 cell apoptosis. Our findings provide potential insights into the interventional treatment of CRC. The specific signaling pathways involved in ROS-induced apoptosis need to be further clarified.

## Data Availability

The article data used to support the findings of this study are available from the corresponding author upon request.

## Supplementary Materials

Supplementary Materials The supplementary file demonstrated viability of Ncm460 cells after treatment with different dose of LCA to evaluated the toxicity of LCA for the normal human colon epithelial cell line.
