# Supplementary material for: Licochalcone a Induces ROS-Mediated Apoptosis through TrxR1 Inactivation in Colorectal Cancer Cells
Source: Biomed Res Int. 2020 May 27;2020:5875074. doi: 10.1155/2020/5875074 (PMC7275230; doi:10.1155/2020/5875074)
Supplement: Supplementary Materials — The supplementary file demonstrated viability of Ncm460 cells after treatment with different dose of LCA to evaluated the toxicity of LCA for the normal human colon epithelial cell line. [file 5875074.f1.pdf]

Licochalcone A induces ROS-mediated apoptosis through TrxR1 inactivation in colorectal cancer cells

Running title: LCA induces apoptosis via TrxR1 inactivation

Peng Wu [1, #], Ting Yu [2, #], Jun Wu [3], Junfeng Chen [4,*]

[1]*Department of Clinical Laboratory, Renmin Hospital of Wuhan University, Wuhan 430060, China*
[2]*Wuhan Mental Health Center, Wuhan 430012, China*
[3]*Department of Pharmacy, Wuhan Jinyin Tan Hospital, Wuhan 430023, China*
[4]*Department of Clinical Laboratory, HuaMei Hospital, University of Chinese Academy of Sciences, Ningbo 315010, China*

[#] The two authors contributed equally to this work.

*Corresponding author. chen861206@163.com

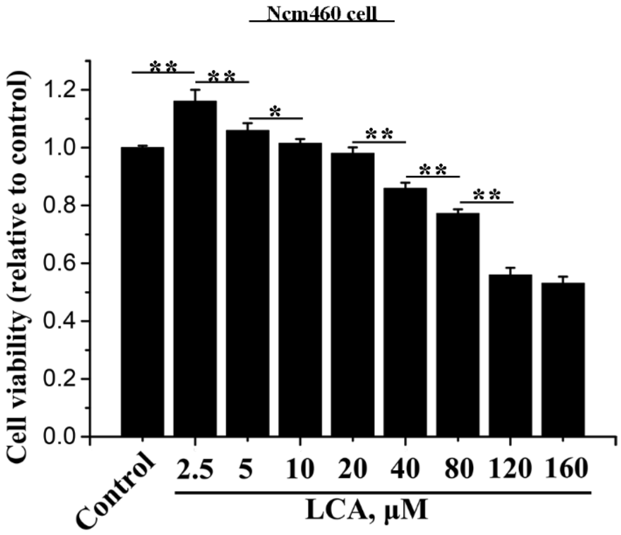

Figure S1. Viability of Ncm460 cells after treatment with different doses of LCA was measured using MTT assay. The results demonstrated that the survival of cells treated with LCA (40 μM) for 24 h remained at over 80% of that of the control cells, indicating the minimal toxicity of LCA on Ncm460 cells.
